# Development and validation of a routine urinalysis-based risk stratification model for IgA nephropathy in a multicenter real-world hospital population

**DOI:** 10.3389/fneph.2026.1826486

**Published:** 2026-08-11

**Authors:** Chi Wang, Wan He, QingYuan Zheng, Yuan Zhong, Meng Tang, ChunYing Zhang, Yong He

**Affiliations:** 1 Department of Laboratory Medicine, West China Hospital, Sichuan University, Chengdu, China; 2 Sichuan Clinical Research Center for Laboratory Medicine, Chengdu, China; 3 Clinical Laboratory Medicine Research Center of West China Hospital, Chengdu, China

**Keywords:** IgA nephropathy, LASSO regression, prediction model, real-world data, risk stratification, routine urinalysis

## Abstract

**Background:**

IgA nephropathy (IgAN) is the most common primary glomerular disease worldwide. Routine urinalysis is universally accessible but underutilized for IgAN risk stratification. This study aimed to develop and validate a urinalysis-based risk stratification model for IgAN in a multicenter real-world hospital population.

**Methods:**

This retrospective multicenter study was based on routine urinalysis records from 8 hospitals. A total of 354 patients had biopsy-confirmed primary IgAN, and 18,002 controls had no documented IgAN diagnosis after electronic medical record (EMR) review. The dataset was randomly divided into a training cohort (70%) and a hold-out validation cohort (30%). LASSO penalized logistic regression was used for variable selection. Model performance was evaluated by discrimination, calibration, and decision curve analysis (DCA). Bootstrap validation with 1000 resamplings was performed. An 8-variable simplified model was additionally developed.

**Results:**

LASSO regression selected 13 variables for the primary full model. The primary model achieved an AUC of 0.849 (95% CI: 0.824–0.873) in the training cohort and 0.861 (95% CI: 0.833–0.890) in the validation cohort. Calibration was good in the training cohort (Brier score = 0.017), while moderate calibration deviation was observed in the validation cohort (Brier score = 0.020). The model yielded a high negative predictive value (>0.99). The 8-variable simplified model retained favorable discrimination with a validation AUC of 0.840.

**Conclusions:**

This routine urinalysis-based model may serve as an accessible laboratory-based risk stratification tool for identifying patients with relatively low or elevated predicted probability of IgAN in real-world hospital settings. It should be interpreted as an adjunct to clinical evaluation rather than as a stand-alone diagnostic test or replacement for renal biopsy.

## 1 Introduction

Immunoglobulin A nephropathy (IgAN), first described by Berger and Hinglais in 1968, is the most prevalent primary glomerular disease worldwide and a leading cause of end-stage renal disease (ESRD) among young and middle-aged adults (1, 2). Characterized by predominant IgA1 deposition in the glomerular mesangium with mesangial cell proliferation and matrix expansion, IgAN exhibits substantial clinical and pathological heterogeneity (3, 4). Approximately 20-30% of patients progress to ESRD within 10–20 years following diagnosis, highlighting the importance of early clinical recognition and risk assessment (5).

The gold standard for IgAN confirmation remains renal biopsy, a procedure that is invasive, technically demanding, and associated with potential complications (e.g., bleeding), particularly in patients with bleeding tendencies or skin infections (6, 7). Additionally, while galactose-deficient IgA1 (Gd-IgA1)-specific antiglycan antibodies show promise as diagnostic markers, their clinical application is not yet widespread (8). The Kidney Disease Improving Global Outcomes (KDIGO) guidelines acknowledge the lack of reliable serum or urinary screening tools for IgAN, highlighting an unmet clinical need for non-invasive, accessible diagnostic tools (9, 10).

Urinalysis represents one of the most commonly performed laboratory examinations in clinical medicine. Unlike other biological fluids regulated by homeostatic mechanisms, urine serves as a reservoir that reflects and accumulates alterations in internal homeostasis, making it particularly suitable for detecting early disease-related changes (11, 12). Urine contains abundant metabolic products that provide valuable information regarding physiological status and disease manifestation, facilitating screening of renal diseases, urinary tract infections, and metabolic disorders (12, 13). Despite its widespread use, the composite predictive value of routine urinalysis parameters for IgAN risk stratification remains underexplored. Previous studies have largely focused on isolated markers or limited clinical cohorts, lacking the scale and methodological rigor required for real-world implementation, highlighting the importance of early clinical recognition and risk assessment (14, 15).

Therefore, this multicenter hospital laboratory-based study aimed to develop and validate a routine urinalysis-based risk stratification model for IgAN. The model was designed to support laboratory-based identification of patients with relatively elevated predicted probability of IgAN in real-world hospital urinalysis settings, rather than to provide a stand-alone diagnosis or replace renal biopsy.

## 2 Materials and methods

### 2.1 Study design and population

This was a multicenter cross-sectional study conducted among outpatients and inpatients from 8 hospitals (West China Hospital of Sichuan University, Shanghai Fengxian District Central Hospital, Shanghai General Hospital, The First Hospital of Jilin University, University-Town Affiliated Hospital of Chongqing Medical University, West China Second University Hospital, Sichuan University, The Second Affiliated Hospital of Kunming Medical University and The First Affiliated Hospital of Henan University of CM). The study period was from October 2023 to December 2025. During routine clinical practice, urine specimens from outpatients and inpatients were submitted to the laboratory for standard urinalysis, and the corresponding results were recorded in the laboratory information system.

Urinalysis records were linked with EMRs using unique patient identifiers. IgAN cases were defined as patients with biopsy-confirmed primary IgAN, as determined by renal pathology reports and EMR review. Controls were defined as patients who underwent routine urinalysis during the same period and had no documented diagnosis of IgAN after review of EMRs, diagnostic records, nephrology records, and available pathology reports. When multiple urinalysis records were available for the same patient, one eligible index record was retained to avoid repeated measurements from the same individual. When available, urinalysis records obtained before disease-specific treatment were preferentially selected for biopsy-confirmed IgAN cases. This study was approved by the institutional review board, and the requirement for informed consent was waived.

### 2.2 Data collection and laboratory measurements

Urine samples were collected from eligible participants during outpatient visits or hospitalization. All samples underwent standardized routine urinalysis using the CA-500 Automatic Urine Analyzer and MA-500 Automated Urine Sediment Analyzer (MEDICSIDE Corp, China), ensuring methodological consistency across all participating centers. For samples yielding inconclusive automated results, manual microscopic examination was performed by experienced laboratory technicians to confirm findings. Patients receiving renin-angiotensin-aldosterone system inhibitors (RAASi) or immunosuppressive agents prior to urinalysis were excluded to prevent confounding from treatment-modified urinalysis profiles. A total of 50 routine urine test variables were recorded, including:

Dipstick parameters: Urobilinogen (UBG), bilirubin (BIL), ketone bodies (KET), occult blood (BLD), protein (PRO), nitrite (NIT), leukocyte esterase (LEU), glucose (GLU), pH, vitamin C (VC), microalbumin (MALB), creatinine (Cr), urinary calcium (CA), albumin/creatinine ratio (A:C), specific gravity (SG), conductivity (Cond), osmolality (Osm).

Urinary sediment quantitative parameters: Red blood cells (RBC), normal RBC, microcytes, acanthocytes, ghost cells, other abnormal RBCs, leukocytes, leukocyte clusters, squamous epithelial cells, non-squamous epithelial cells, renal tubular epithelial cells, caudate epithelial cells, small round epithelial cells, hyaline casts, pathological casts, granular casts, waxy casts, broad casts, other casts, bacilli, total yeasts, yeasts, candida, crystals, calcium oxalate monohydrate crystals, calcium oxalate dihydrate crystals, uric acid crystals, magnesium ammonium phosphate crystals, amorphous salt crystals, other crystals, sperm, mucus threads, suspected cocci.

Laboratory measurements were performed according to CLSI guidelines and the operational manuals of the analyzers. Dipstick semi-quantitative results were converted to ordinal variables for statistical analysis (0 = negative, 1 = 1+, 2 = 2+, 3 = 3+, 4 = 4+, 5 = 5+), while urinary sediment parameters were used directly as continuous variables (reported as counts per high-power field [HPF] or low-power field [LPF]).

### 2.3 Statistical analysis

#### 2.3.1 Data preprocessing and standardization

The dataset was randomly divided into a training cohort (70%) and a hold-out validation cohort (30%). Continuous variables were standardized using z-scores to improve coefficient interpretability and numerical stability. The mean and standard deviation used for standardization were estimated exclusively from the training cohort and then applied unchanged to the hold-out validation cohort to avoid information leakage.

#### 2.3.2 Missing data handling

Missing values were observed only for “coccilike bacteria” (**Supplementary Table 2**), with 35 missing observations (0.19% of this variable, ~0.004% of all data cells). Median imputation was applied. This variable was not retained by LASSO and was not included in the final model.

#### 2.3.3 LASSO variable selection

LASSO-penalized logistic regression with 10-fold cross-validation was employed (16). The optimal penalty parameter λ was selected using the one-standard-error (1-SE) rule (λ = 0.002675191). All variables with non-zero coefficients at the selected λ were retained for the final model.

#### 2.3.4 Model construction

Variables selected by LASSO were entered into a multivariable logistic regression model. *P* values were reported for descriptive purposes only; no additional variable elimination was performed based on post-LASSO *P*-values. Variance inflation factor (VIF) was calculated (threshold <5). To improve clinical applicability, an 8-variable simplified model using only dry chemistry parameters was additionally constructed as an implementation-oriented secondary model.

#### 2.3.5 Model validation and performance evaluation

Model performance was evaluated by discrimination (AUC), calibration (Hosmer-Lemeshow test, Brier score, calibration intercept and slope), and clinical utility (DCA). Diagnostic metrics including sensitivity, specificity, PPV, NPV, F1 score, and balanced accuracy were calculated. Bootstrap validation with 1000 resamplings was performed to estimate AUC stability. All analyses were performed using R software (version 4.3.1).

## 3 Results

### 3.1 Baseline characteristics

**Figure 1:**
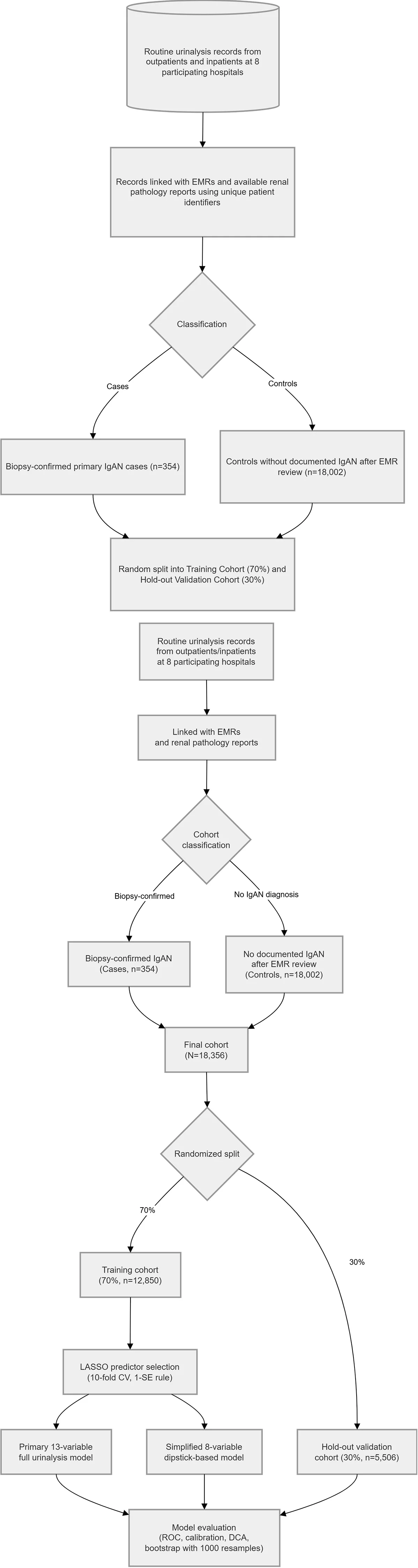
Study flow diagram of the multicenter hospital laboratory-based routine urinalysis cohort. Routine urinalysis records were collected from outpatients and inpatients at eight participating hospitals. Urinalysis records were linked with electronic medical records and available renal pathology reports. IgAN cases were defined as patients with biopsy-confirmed primary IgAN, whereas controls were defined as patients without documented IgAN diagnosis after EMR review. The final cohort included 354 biopsy-confirmed IgAN cases and 18,002 controls without documented IgAN diagnosis. The cohort was randomly divided into a training cohort and a hold-out validation cohort. EMR, electronic medical record; IgAN, IgA nephropathy; LASSO, least absolute shrinkage and selection operator.

Study participants were enrolled and processed as outlined in **Figure 1**. A total of 18,356 hospital-based patients undergoing routine urinalysis were included, comprising 354 biopsy-confirmed IgAN cases and 18,002 controls without documented IgAN diagnosis after EMR review. The training cohort included 12,850 patients, of whom 230 had biopsy-confirmed IgAN, whereas the hold-out validation cohort included 5,506 patients, of whom 124 had biopsy-confirmed IgAN. Key urinalysis characteristics related to the final models are summarized in **Table 1**, and the full distribution of all 50 candidate urinalysis variables is provided in **Supplementary Table 1**.

**Table 1:** Key urinalysis characteristics related to the final models according to IgAN status.

Characteristic	Total cohort (N = 18,356)	Controls without documented IgAN (N = 18,002)	Biopsy-confirmed IgAN (N = 354)	*P* value
Outcome group				
Participants, n (%)	18,356 (100.00)	18,002 (98.07)	354 (1.93)	—
Dipstick-based predictors				
Occult blood, grade, median [IQR]	0 [0; 1]	0 [0; 1]	1 [0; 3]	<0.001
Protein, grade, median [IQR]	0 [0; 2]	0 [0; 2]	3 [1; 3]	<0.001
Leukocyte esterase, grade, median [IQR]	0 [0; 1]	0 [0; 1]	0 [0; 0]	<0.001
Glucose, grade, median [IQR]	0 [0; 0]	0 [0; 0]	0 [0; 3]	<0.001
Ketone bodies, grade, median [IQR]	0 [0; 0]	0 [0; 0]	0 [0; 0]	0.005
pH, median [IQR]	5.5 [5.5; 6.0]	5.5 [5.5; 6.5]	5.5 [5.0; 6.0]	<0.001
Vitamin C, grade, median [IQR]	0 [0; 1]	0 [0; 1]	1 [0; 1]	<0.001
Specific gravity, median [IQR]	1.02 [1.01; 1.02]	1.02 [1.01; 1.02]	1.02 [1.01; 1.02]	0.436
Additional predictors in the primary 13-variable model				
Microalbumin, grade, median [IQR]	1 [0; 3]	1 [0; 3]	3 [3; 3]	<0.001
Creatinine, grade, median [IQR]	2 [1; 3]	2 [1; 3]	3 [2; 3]	<0.001
Squamous epithelial cells, median [IQR]	1.00 [0.00; 2.00]	1.00 [0.00; 2.00]	1.00 [0.00; 3.00]	0.437
Conductivity, mS/cm, median [IQR]	16.60 [11.90; 21.70]	16.60 [11.90; 21.80]	13.90 [9.70; 18.85]	<0.001
Acanthocytes, median [IQR]	0.00 [0.00; 0.00]	0.00 [0.00; 0.00]	0.00 [0.00; 1.00]	<0.001

Values are presented as median [interquartile range] unless otherwise indicated. Dipstick semi-quantitative parameters were coded as ordinal grades. *P* values compare biopsy-confirmed IgAN cases and controls without documented IgAN diagnosis. Mann-Whitney U test was used for continuous or ordinal variables, and chi-square test was used for categorical variables. IgAN, IgA nephropathy; IQR, interquartile range.

### 3.2 Predictor selection

**Figure 2:**
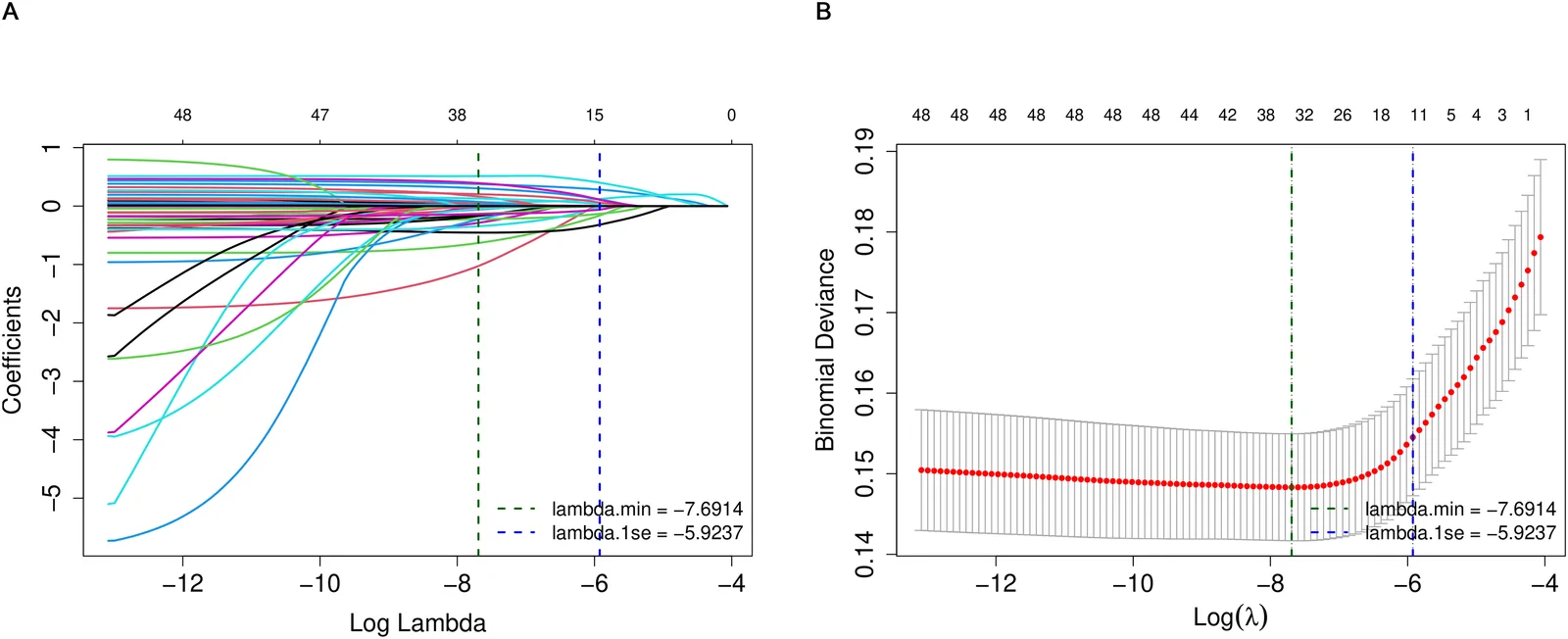
Predictor selection using LASSO penalized logistic regression. **(A)** LASSO coefficient profiles of the 50 candidate routine urinalysis variables. **(B)** Ten-fold cross-validation curve for selection of the penalty parameter λ. Thirteen variables with non-zero coefficients at the selected λ (λ = 0.002675191) were retained for the primary full urinalysis model. The vertical dotted lines indicate λmin and λ1se. LASSO, least absolute shrinkage and selection operator.

Among the 50 candidate variables, LASSO regression retained 13 variables for the primary full urinalysis model (**Figure 2**; **Table 2**). The events per variable (EPV) was 17.7 for the 13-variable model and 28.8 for the 8-variable model.

**Table 2:** Post-LASSO logistic regression coefficients of the primary 13-variable full urinalysis model.

Variable	β	SE	OR	95% CI	*P* value	VIF
Intercept	-5.513	0.76	0.004	0.001–0.018	<0.001	–
Microalbumin	0.629	0.096	1.876	1.555–2.264	<0.001	1.676
Creatinine	0.426	0.078	1.531	1.313–1.784	<0.001	1.544
Occult blood	0.369	0.046	1.446	1.321–1.584	<0.001	1.23
Vitamin C	0.332	0.077	1.394	1.199–1.620	<0.001	1.077
Glucose	0.239	0.043	1.27	1.168–1.382	<0.001	1.462
Squamous epithelial cells	0.129	0.04	1.137	1.052–1.229	0.001	1.037
Acanthocytes	0.018	0.02	1.018	0.978–1.060	0.387	1.045
Protein	0.027	0.065	1.027	0.904–1.167	0.683	1.813
Ketone bodies	-0.843	0.27	0.43	0.253–0.731	0.002	1.04
Leukocyte esterase	-0.624	0.095	0.536	0.445–0.645	<0.001	1.096
Specific gravity	-0.442	0.103	0.643	0.526–0.786	<0.001	1.803
pH	-0.253	0.116	0.776	0.619–0.973	0.028	1.076
Conductivity	-0.152	0.085	0.859	0.728–1.014	0.073	1.366

Continuous variables were standardized using z-scores before model fitting; odds ratios for standardized continuous variables correspond to a 1-standard-deviation increase. Semi-quantitative dipstick variables were entered as ordinal variables. *P* values are provided for descriptive purposes only and were not used for additional variable elimination after LASSO selection. VIF, variance inflation factor. The LASSO penalty parameter used was λ = 0.002675191.

### 3.3 Performance of the primary 13-variable model

#### 3.3.1 Discrimination

**Figure 3:**
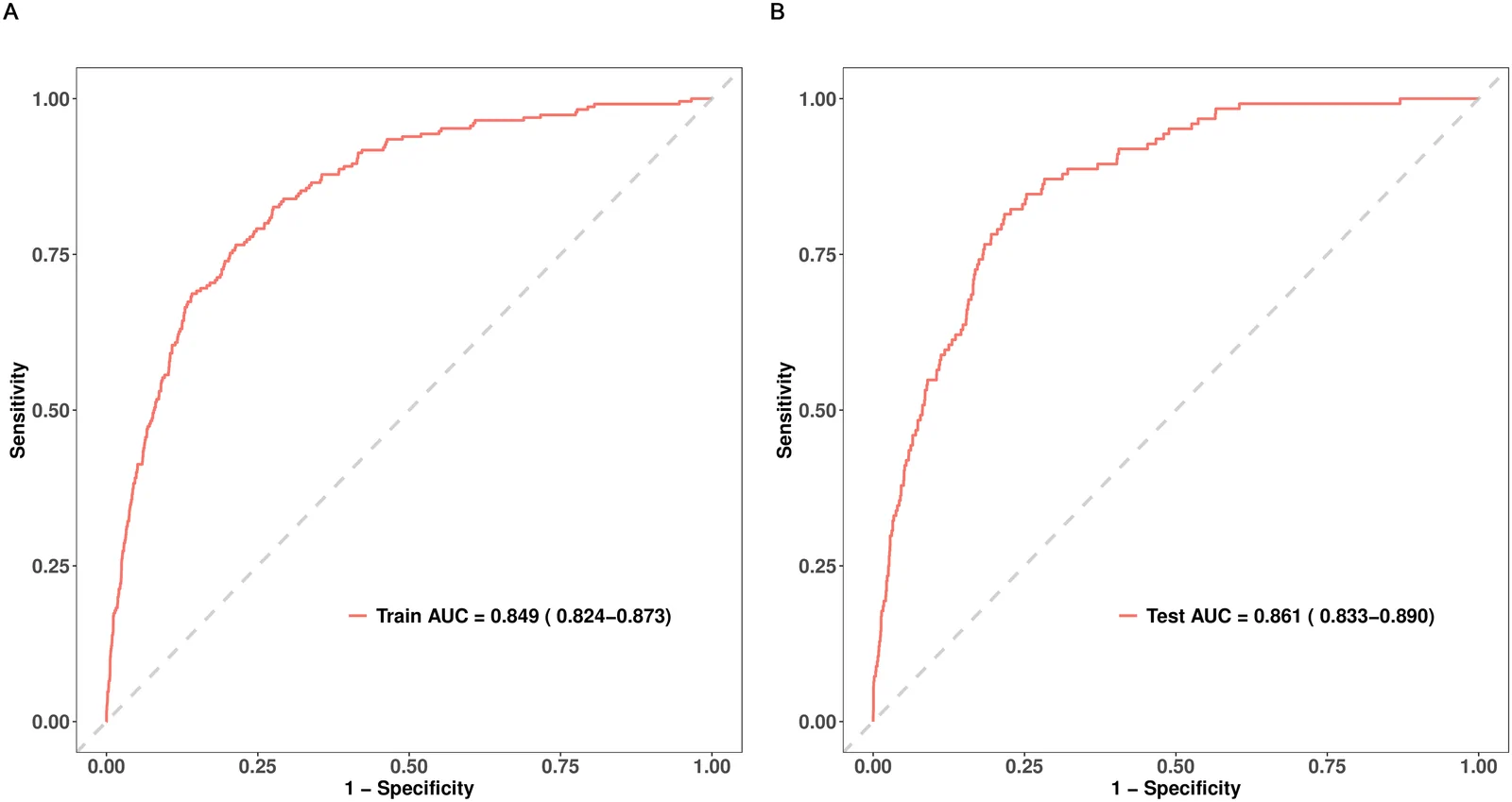
Receiver operating characteristic curves of the primary 13-variable full urinalysis model. **(A)** Training cohort: AUC = 0.849, 95% CI: 0.824–0.873. **(B)** Hold-out validation cohort: AUC = 0.861, 95% CI: 0.833–0.890. AUC, area under the receiver operating characteristic curve; CI, confidence interval.

The primary 13-variable model showed an AUC of 0.849 (95% CI: 0.824–0.873) in the training cohort and 0.861 (95% CI: 0.833–0.890) in the hold-out validation cohort (**Figure 3**). All VIF values were below 5.0.

#### 3.3.2 Calibration

**Figure 4:**
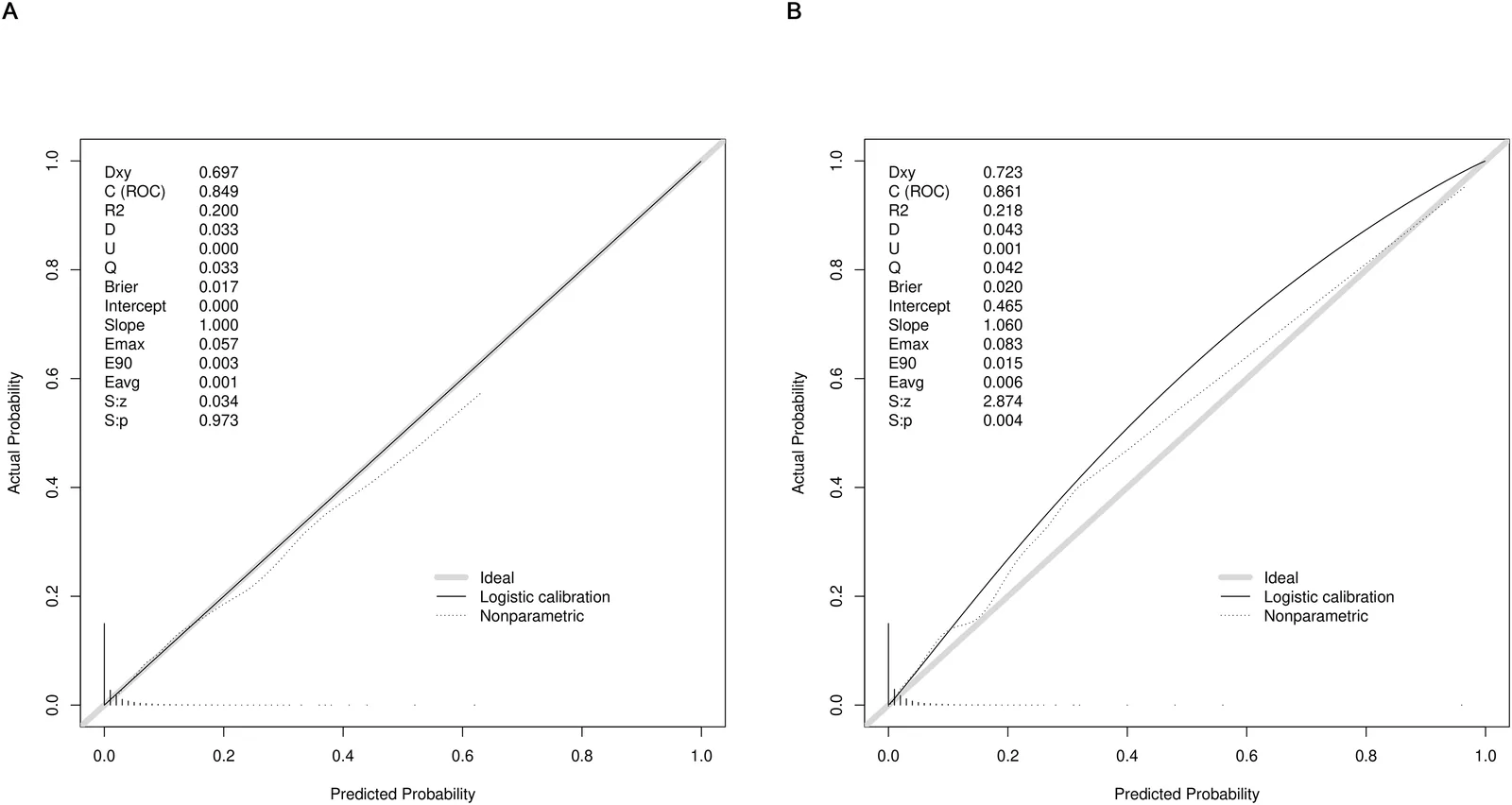
Calibration curves of the primary 13-variable full urinalysis model. **(A)** Training cohort. **(B)** Hold-out validation cohort. The diagonal line represents ideal calibration. The primary model showed excellent calibration in the training cohort, whereas some calibration deviation was observed in the validation cohort despite a low Brier score.

Calibration was good in the training cohort (Brier score = 0.017; Hosmer-Lemeshow *P* = 0.649; intercept = 0.000; slope = 1.000). In the validation cohort, the Brier score remained low (0.020), but the Hosmer-Lemeshow test reached statistical significance (*P* = 0.017), indicating that the model is more suitable for relative risk stratification than for precise estimation of absolute probability (**Figure 4**).

#### 3.3.3 Clinical utility

**Figure 5:**
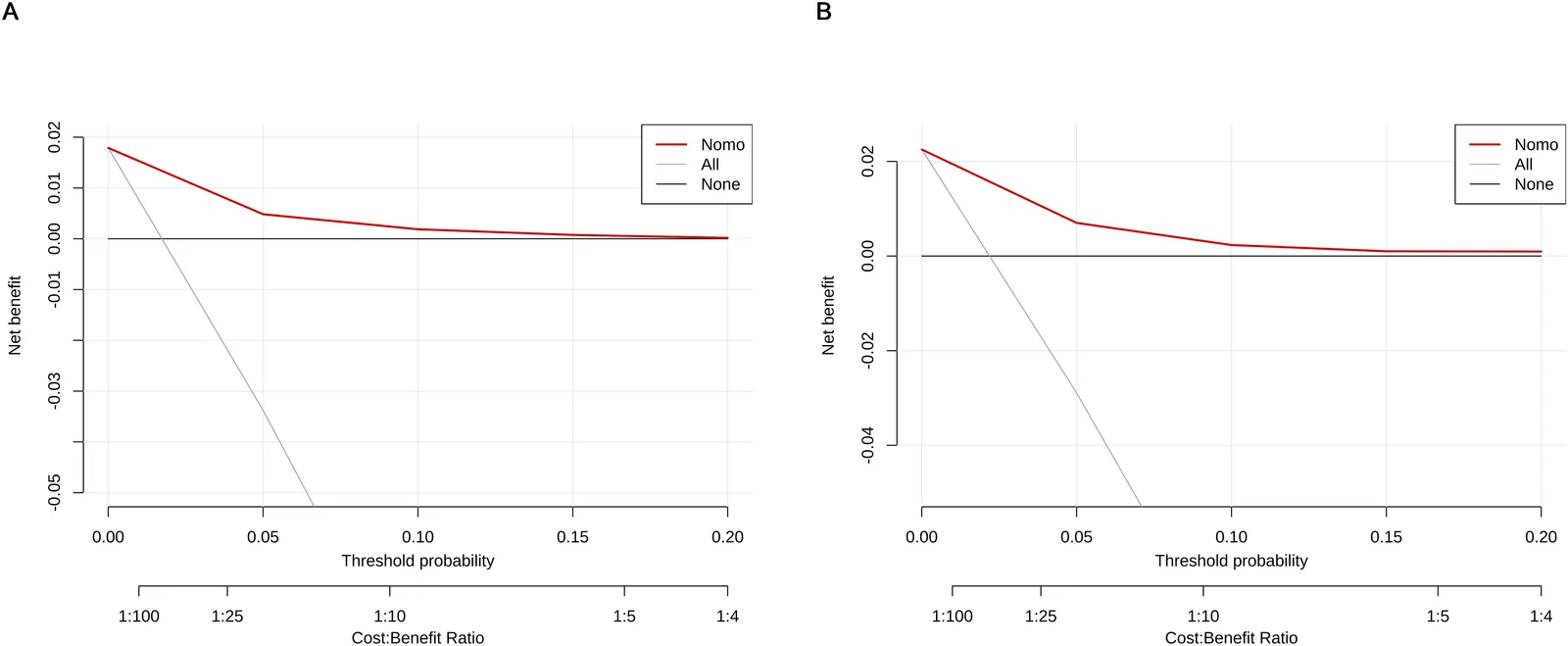
Decision curve analysis of the primary 13-variable full urinalysis model. **(A)** Training cohort. **(B)** Hold-out validation cohort. Decision curves were displayed across threshold probabilities from 0 to 0.20 because IgAN prevalence was low in this hospital-based urinalysis cohort and low threshold probabilities were considered more relevant for laboratory-based risk stratification. DCA, decision curve analysis; IgAN, IgA nephropathy.

Decision curve analysis (DCA) showed that the primary 13-variable model provided a positive net benefit compared with the treat-all and treat-none strategies mainly within the low-threshold probability range from 0 to 0.20 (**Figure 5**).

#### 3.3.4 Diagnostic performance

The model achieved a high negative predictive value (NPV > 0.99) in both cohorts, while the positive predictive value (PPV) was low (~6-8%) due to the low population prevalence of IgAN (**Table 3**).

**Table 3:** Performance metrics of the primary 13-variable full model.

Metric	Training cohort	Validation cohort
Discrimination		
AUC	0.849	0.861
95% CI (DeLong)	0.824–0.873	0.833–0.890
Bootstrap AUC (1000 resamples)	0.841	0.857
Calibration		
Brier score	0.017	0.020
Calibration intercept	0.000	0.465
Calibration slope	1.000	1.060
Hosmer-Lemeshow P value	0.649	0.017
Diagnostic performance		
Sensitivity	0.765	0.790
Specificity	0.787	0.794
Positive predictive value	0.061	0.081
Negative predictive value	0.995	0.994

### 3.4 Simplified 8-variable model

To further address clinical applicability, we developed a simplified dipstick-based model using eight routinely available parameters: occult blood, protein, leukocyte esterase, glucose, ketone bodies, pH, vitamin C, and specific gravity. The simplified model showed favorable discrimination, with an AUC of 0.827 in the training cohort and 0.840 in the validation cohort (**Supplementary Figure 2**; **Supplementary Table 4**). Calibration was good in the training cohort (Brier = 0.017, Hosmer-Lemeshow *P* = 0.3135), whereas less consistent calibration was observed in the validation cohort despite a low Brier score (Brier = 0.021, Hosmer-Lemeshow *P* = 0.0004). Therefore, this model was considered an implementation-oriented secondary model rather than a replacement for the primary full model (**Table 4**; **Supplementary Figure 3**).

**Table 4:** Performance metrics of the simplified 8-variable model.

Metric	Training cohort	Validation cohort
Discrimination		
AUC	0.827	0.840
95% CI (DeLong)	0.802–0.852	0.811–0.869
Bootstrap AUC (1000 resamples)	0.821	0.840
Calibration		
Brier score	0.017	0.021
Hosmer-Lemeshow P value	0.3135	0.0004
Diagnostic performance		
Sensitivity	0.770	0.750
Specificity	0.767	0.775
Positive predictive value	0.057	0.071
Negative predictive value	0.995	0.993
F1 score	0.106	0.130
Balanced accuracy	0.768	0.762

### 3.5 Bootstrap internal validation

Bootstrap validation with 1000 resamplings yielded comparable AUC estimates (0.841 in training, 0.857 in validation), suggesting no obvious evidence of severe overfitting (**Supplementary Figure 1**; **Supplementary Table 2**).

## 4 Discussion

### 4.1 Principal findings

This multicenter laboratory-based urinalysis study developed a 13-variable full model and an 8-variable simplified implementation model for IgAN risk stratification in a real-world hospital setting.

### 4.2 Clinical positioning and intended use

It is imperative to emphasize that this model should be used for laboratory-based risk stratification rather than stand-alone diagnosis or rule-out decision-making. Due to the low prevalence of IgAN, the model yields a low PPV but a high NPV (>0.99). The high NPV should be interpreted in the context of low disease prevalence. It may help identify patients with relatively low predicted probability, but it should not be used alone to exclude IgAN when clinical suspicion remains high.

### 4.3 Interpretation of predictors

Classic IgAN-related markers (occult blood, microalbumin) reflect glomerular injury (17, 18). Non-classical variables (vitamin C, ketone bodies, specific gravity) may reflect urine concentration, metabolic status, tubular handling, or comorbid conditions, and their inclusion should be interpreted primarily as incremental predictive information rather than direct mechanistic evidence (6, 19).

### 4.4 Clinical applicability of the simplified model

The simplified model retained favorable discrimination and may support implementation in settings where only commonly available dipstick urinalysis parameters are available. However, validation calibration was less consistent, so absolute probabilities should be interpreted cautiously.

### 4.5 Limitations

First, controls were defined by the absence of documented IgAN rather than universal biopsy verification, which inherently introduces verification bias and may include occult IgAN cases. Second, the low prevalence of IgAN resulted in a low PPV. Third, the model cannot be used to independently rule out IgAN. Fourth, statistically significant calibration deviation was observed in the validation cohort for both models. Fifth, clinical variables (age, sex, BP, eGFR) were not incorporated. Sixth, exclusion of patients receiving RAAS inhibitors or immunosuppressive therapy may reduce confounding from treatment-modified urinalysis profiles, but it may also limit generalizability to treated populations. Although a hold-out validation cohort and bootstrap validation were used, both were derived from the same multicenter retrospective dataset; therefore, the present study does not constitute prospective external validation. Prospective external validation with standardized diagnostic follow-up is warranted.

## 5 Conclusion

In this multicenter hospital laboratory-based routine urinalysis cohort, we developed and validated a 13-variable LASSO-selected full urinalysis model for IgAN risk stratification. The model showed favorable discrimination and low overall prediction error, although calibration deviation was observed in the validation cohort. An 8-variable simplified dipstick-based model retained acceptable discrimination and may support implementation in settings where comprehensive urinalysis parameters are unavailable. These models should be interpreted as adjunctive risk stratification tools and should not replace clinical evaluation or renal biopsy.

## Data Availability

The original contributions presented in the study are included in the article/**Supplementary Material**. Further inquiries can be directed to the corresponding authors.
