# Supplementary material for: Development and validation of a routine urinalysis-based risk stratification model for IgA nephropathy in a multicenter real-world hospital population
Source: Front Nephrol. 2026 Aug 11;6:1826486. doi: 10.3389/fneph.2026.1826486 (PMC13503150; doi:10.3389/fneph.2026.1826486)
Supplement: Supplementary file 1 [file Table1.docx]

**Supplementary Figure S1. Bootstrap ROC curves of the primary 13-variable full urinalysis model.**

(A) Training cohort. (B) Hold-out validation cohort. Bootstrap validation was performed using 1000 resamplings. The red line represents the apparent ROC curve, and the gray lines represent bootstrap ROC curves.
