# Supplementary material for: Development and validation of a routine urinalysis-based risk stratification model for IgA nephropathy in a multicenter real-world hospital population
Source: Front Nephrol. 2026 Aug 11;6:1826486. doi: 10.3389/fneph.2026.1826486 (PMC13503150; doi:10.3389/fneph.2026.1826486)
Supplement: Supplementary file 2 [file Table2.docx]

**Supplementary Figure S2. ROC curves of the simplified 8-variable dipstick-based model.**

(A) Training cohort: AUC = 0.827, 95% CI: 0.802–0.852. (B) Hold-out validation cohort: AUC = 0.840, 95% CI: 0.811–0.869.
