# Supplementary material for: Development and validation of a routine urinalysis-based risk stratification model for IgA nephropathy in a multicenter real-world hospital population
Source: Front Nephrol. 2026 Aug 11;6:1826486. doi: 10.3389/fneph.2026.1826486 (PMC13503150; doi:10.3389/fneph.2026.1826486)
Supplement: Supplementary file 3 [file Table3.docx]

**Supplementary Figure S3. Calibration curves of the simplified 8-variable dipstick-based model.**

(A) Training cohort. (B) Hold-out validation cohort. The simplified model showed good calibration in the training cohort, whereas less consistent calibration was observed in the validation cohort despite a low Brier score.
