# Supplementary material for: Development and validation of a routine urinalysis-based risk stratification model for IgA nephropathy in a multicenter real-world hospital population
Source: Front Nephrol. 2026 Aug 11;6:1826486. doi: 10.3389/fneph.2026.1826486 (PMC13503150; doi:10.3389/fneph.2026.1826486)
Supplement: Supplementary file 4 [file Table4.docx]

**Table S1. Distribution of all 50 candidate routine urinalysis variables according to IgAN status**

| **Variables** | **Total**  **(N=18,356)** | **Controls without documented IgAN (N=18,002)** | **Biopsy-confirmed IgAN (N=354)** | ***P* Value** |
| --- | --- | --- | --- | --- |
| **Physical and Quantitative Parameters** |  |  |  |  |
| Specific Gravity (SG) | 1.02 [1.01; 1.02] | 1.02 [1.01; 1.02] | 1.02 [1.01; 1.02] | 0.436 |
| Conductivity (mS/cm) | 16.60 [11.90; 21.70] | 16.60 [11.90; 21.80] | 13.90 [9.70; 18.85] | <0.001 |
| Osmolality (mOsm/kg) | 512.35 [394.85; 634.55] | 514.70 [397.20; 634.55] | 446.55 [367.24; 562.88] | <0.001 |
| Sediment Analysis (cells/HPF) |  |  |  |  |
| Red Blood Cells (RBC) | 3.00 [2.00; 5.00] | 3.00 [2.00; 5.00] | 8.00 [3.00; 26.00] | <0.001 |
| Normomorphic RBC | 0.00 [0.00; 1.00] | 0.00 [0.00; 1.00] | 1.00 [0.00; 4.00] | <0.001 |
| Microcytic RBC | 0.00 [0.00; 1.00] | 0.00 [0.00; 1.00] | 1.00 [0.00; 2.00] | <0.001 |
| Acanthocytes | 0.00 [0.00; 0.00] | 0.00 [0.00; 0.00] | 0.00 [0.00; 1.00] | <0.001 |
| Ghost Cells | 1.00 [0.00; 1.00] | 1.00 [0.00; 1.00] | 1.00 [1.00; 3.00] | <0.001 |
| Other Dysmorphic RBCs | 1.00 [1.00; 2.00] | 1.00 [1.00; 2.00] | 3.00 [1.00; 14.75] | <0.001 |
| White Blood Cells (WBC) | 2.00 [1.00; 6.00] | 2.00 [1.00; 6.00] | 3.00 [2.00; 6.00] | 0.005 |
| WBC Clumps | 0.00 [0.00; 0.00] | 0.00 [0.00; 0.00] | 0.00 [0.00; 0.00] | <0.001 |
| Squamous Epithelial Cells | 1.00 [0.00; 2.00] | 1.00 [0.00; 2.00] | 1.00 [0.00; 3.00] | 0.437 |
| Nonsquamous Epithelial Cells | 0.00 [0.00; 1.00] | 0.00 [0.00; 1.00] | 0.00 [0.00; 1.00] | <0.001 |
| Renal Tubular Epithelial Cells | 0.00 [0.00; 0.00] | 0.00 [0.00; 0.00] | 0.00 [0.00; 0.00] | <0.001 |
| Caudate Epithelial Cells | 0.00 [0.00; 0.00] | 0.00 [0.00; 0.00] | 0.00 [0.00; 0.00] | 0.861 |
| Small Round Epithelial Cells | 0.00 [0.00; 1.00] | 0.00 [0.00; 1.00] | 0.00 [0.00; 0.00] | <0.001 |
| **Casts and Other Elements** |  |  |  |  |
| Hyaline Casts (casts/LPF) | 0.00 [0.00; 0.00] | 0.00 [0.00; 0.00] | 0.00 [0.00; 2.00] | <0.001 |
| Pathologic Casts (casts/LPF) | 0.00 [0.00; 0.00] | 0.00 [0.00; 0.00] | 0.00 [0.00; 1.00] | <0.001 |
| Granular Casts (casts/LPF) | 0.00 [0.00; 0.00] | 0.00 [0.00; 0.00] | 0.00 [0.00; 1.00] | <0.001 |
| Waxy Casts (casts/LPF) | 0.00 [0.00; 0.00] | 0.00 [0.00; 0.00] | 0.00 [0.00; 0.00] | 0.469 |
| Broad Casts (casts/LPF) | 0.00 [0.00; 0.00] | 0.00 [0.00; 0.00] | 0.00 [0.00; 0.00] | 0.51 |
| Other Casts (casts/LPF) | 0.00 [0.00; 0.00] | 0.00 [0.00; 0.00] | 0.00 [0.00; 0.00] | 0.002 |
| Bacilli (units/HPF) | 1.00 [1.00; 4.00] | 1.00 [1.00; 4.00] | 1.00 [1.00; 11.00] | <0.001 |
| Yeast (Total) (units/HPF) | 0.00 [0.00; 0.00] | 0.00 [0.00; 0.00] | 0.00 [0.00; 0.00] | 0.165 |
| Yeastlike Cells (units/HPF) | 0.00 [0.00; 0.00] | 0.00 [0.00; 0.00] | 0.00 [0.00; 0.00] | 0.205 |
| Candida (units/HPF) | 0.00 [0.00; 0.00] | 0.00 [0.00; 0.00] | 0.00 [0.00; 0.00] | 0.122 |
| Crystals (Total) (units/HPF) | 0.00 [0.00; 0.00] | 0.00 [0.00; 0.00] | 0.00 [0.00; 0.00] | <0.001 |
| CaOxalate Monohydrate (units/HPF) | 0.00 [0.00; 0.00] | 0.00 [0.00; 0.00] | 0.00 [0.00; 0.00] | 0.56 |
| CaOxalate Dihydrate (units/HPF) | 0.00 [0.00; 0.00] | 0.00 [0.00; 0.00] | 0.00 [0.00; 0.00] | <0.001 |
| Uric Acid Crystals (units/HPF) | 0.00 [0.00; 0.00] | 0.00 [0.00; 0.00] | 0.00 [0.00; 0.00] | 0.173 |
| MgAmmonium Phosphate (units/HPF) | 0.00 [0.00; 0.00] | 0.00 [0.00; 0.00] | 0.00 [0.00; 0.00] | 0.271 |
| Amorphous Salts (units/HPF) | 0.00 [0.00; 0.00] | 0.00 [0.00; 0.00] | 0.00 [0.00; 0.00] | 0.064 |
| Other Crystals (units/HPF) | 0.00 [0.00; 0.00] | 0.00 [0.00; 0.00] | 0.00 [0.00; 0.00] | 0.269 |
| Spermatozoa (units/HPF) | 0.00 [0.00; 0.00] | 0.00 [0.00; 0.00] | 0.00 [0.00; 0.00] | 0.273 |
| Mucus Threads (units/HPF) | 2.00 [0.00; 7.00] | 2.00 [0.00; 7.00] | 1.00 [1.00; 4.00] | 0.133 |
| Coccilike Bacteria (units/HPF) | 0.00 [0.00; 0.00] | 0.00 [0.00; 0.00] | 0.00 [0.00; 0.00] | <0.001 |
| **Semiquantitative Dipstick Parameters** |  |  |  |  |
| Urobilinogen (UBG) |  |  |  | 0.641 |
| 0 | 17,303 (94.26%) | 16,965 (94.24%) | 338 (95.48%) |  |
| 1+ | 1,028 (5.60%) | 1,012 (5.62%) | 16 (4.52%) |  |
| 2+ | 19 (0.10%) | 19 (0.11%) | 0 (0.00%) |  |
| 3+ | 6 (0.03%) | 6 (0.03%) | 0 (0.00%) |  |
| Bilirubin (BIL) |  |  |  | 0.551 |
| 0 | 18,073 (98.46%) | 17,721 (98.44%) | 352 (99.44%) |  |
| 1+ | 239 (1.30%) | 237 (1.32%) | 2 (0.56%) |  |
| 2+ | 38 (0.21%) | 38 (0.21%) | 0 (0.00%) |  |
| 3+ | 6 (0.03%) | 6 (0.03%) | 0 (0.00%) |  |
| Ketones (KET) |  |  |  | 0.005 |
| 0 | 16,608 (90.48%) | 16,270 (90.38%) | 338 (95.48%) |  |
| 1+ | 1,035 (5.64%) | 1,020 (5.67%) | 15 (4.24%) |  |
| 2+ | 414 (2.26%) | 413 (2.29%) | 1 (0.28%) |  |
| 3+ | 242 (1.32%) | 242 (1.34%) | 0 (0.00%) |  |
| 4+ | 42 (0.23%) | 42 (0.23%) | 0 (0.00%) |  |
| 5+ | 15 (0.08%) | 15 (0.08%) | 0 (0.00%) |  |
| Occult Blood (BLD) |  |  |  | <0.001 |
| 0 | 12,575 (68.51%) | 12,451 (69.16%) | 124 (35.03%) |  |
| 1+ | 2,289 (12.47%) | 2,230 (12.39%) | 59 (16.67%) |  |
| 2+ | 1,174 (6.40%) | 1,131 (6.28%) | 43 (12.15%) |  |
| 3+ | 817 (4.45%) | 768 (4.27%) | 49 (13.84%) |  |
| 4+ | 1,501 (8.18%) | 1,422 (7.90%) | 79 (22.32%) |  |
| Protein (PRO) |  |  |  | <0.001 |
| 0 | 10,744 (58.53%) | 10,692 (59.39%) | 52 (14.69%) |  |
| 1+ | 2,788 (15.19%) | 2,737 (15.20%) | 51 (14.41%) |  |
| 2+ | 1,737 (9.46%) | 1,671 (9.28%) | 66 (18.64%) |  |
| 3+ | 1,687 (9.19%) | 1,553 (8.63%) | 134 (37.85%) |  |
| 4+ | 1,365 (7.44%) | 1,314 (7.30%) | 51 (14.41%) |  |
| 5+ | 35 (0.19%) | 35 (0.19%) | 0 (0.00%) |  |
| Nitrite (NIT) |  |  |  | 0.009 |
| 0 | 17,588 (95.82%) | 17,238 (95.76%) | 350 (98.87%) |  |
| 1+ | 64 (0.35%) | 64 (0.36%) | 0 (0.00%) |  |
| 2+ | 704 (3.84%) | 700 (3.89%) | 4 (1.13%) |  |
| Leukocyte Esterase (LEU) |  |  |  | <0.001 |
| 0 | 13,142 (71.60%) | 12,838 (71.31%) | 304 (85.88%) |  |
| 1+ | 1,877 (10.23%) | 1,853 (10.29%) | 24 (6.78%) |  |
| 2+ | 1,262 (6.88%) | 1,247 (6.93%) | 15 (4.24%) |  |
| 3+ | 1,051 (5.73%) | 1,044 (5.80%) | 7 (1.98%) |  |
| 4+ | 1,024 (5.58%) | 1,020 (5.67%) | 4 (1.13%) |  |
| Glucose (GLU) |  |  |  | <0.001 |
| 0 | 14,347 (78.16%) | 14,105 (78.35%) | 242 (68.36%) |  |
| 1+ | 914 (4.98%) | 903 (5.02%) | 11 (3.11%) |  |
| 2+ | 376 (2.05%) | 371 (2.06%) | 5 (1.41%) |  |
| 3+ | 410 (2.23%) | 397 (2.21%) | 13 (3.67%) |  |
| 4+ | 332 (1.81%) | 315 (1.75%) | 17 (4.80%) |  |
| 5+ | 1,977 (10.77%) | 1,911 (10.62%) | 66 (18.64%) |  |
| pH Value |  |  |  | <0.001 |
| 5 | 4,584 (24.97%) | 4,453 (24.74%) | 131 (37.01%) |  |
| 5.5 | 5,343 (29.11%) | 5,230 (29.05%) | 113 (31.92%) |  |
| 6 | 3,860 (21.03%) | 3,806 (21.14%) | 54 (15.25%) |  |
| 6.5 | 2,654 (14.46%) | 2,617 (14.54%) | 37 (10.45%) |  |
| 7 | 1,394 (7.59%) | 1,380 (7.67%) | 14 (3.95%) |  |
| 7.5 | 418 (2.28%) | 414 (2.30%) | 4 (1.13%) |  |
| 8 | 83 (0.45%) | 82 (0.46%) | 1 (0.28%) |  |
| 8.5 | 17 (0.09%) | 17 (0.09%) | 0 (0.00%) |  |
| 9 | 3 (0.02%) | 3 (0.02%) | 0 (0.00%) |  |
| Vitamin C (VC) |  |  |  | <0.001 |
| 0 | 10,842 (59.07%) | 10,711 (59.50%) | 131 (37.01%) |  |
| 1+ | 6,055 (32.99%) | 5,860 (32.55%) | 195 (55.08%) |  |
| 2+ | 767 (4.18%) | 747 (4.15%) | 20 (5.65%) |  |
| 3+ | 391 (2.13%) | 385 (2.14%) | 6 (1.69%) |  |
| 4+ | 301 (1.64%) | 299 (1.66%) | 2 (0.56%) |  |
| Microalbumin (mAlb) |  |  |  | <0.001 |
| 0 | 7,477 (40.73%) | 7,457 (41.42%) | 20 (5.65%) |  |
| 1+ | 2,935 (15.99%) | 2,906 (16.14%) | 29 (8.19%) |  |
| 2+ | 466 (2.54%) | 458 (2.54%) | 8 (2.26%) |  |
| 3+ | 7,418 (40.41%) | 7,121 (39.56%) | 297 (83.90%) |  |
| 4+ | 60 (0.33%) | 60 (0.33%) | 0 (0.00%) |  |
| Creatinine (Cr) |  |  |  | <0.001 |
| 0 | 1,128 (6.15%) | 1,106 (6.14%) | 22 (6.21%) |  |
| 1+ | 3,956 (21.55%) | 3,917 (21.76%) | 39 (11.02%) |  |
| 2+ | 6,085 (33.15%) | 5,987 (33.26%) | 98 (27.68%) |  |
| 3+ | 4,541 (24.74%) | 4,430 (24.61%) | 111 (31.36%) |  |
| 4+ | 2,646 (14.41%) | 2,562 (14.23%) | 84 (23.73%) |  |
| Urine Calcium (Ca) |  |  |  | <0.001 |
| 1+ | 4,827 (26.30%) | 4,711 (26.17%) | 116 (32.77%) |  |
| 2+ | 5,824 (31.73%) | 5,671 (31.50%) | 153 (43.22%) |  |
| 3+ | 7,000 (38.13%) | 6,918 (38.43%) | 82 (23.16%) |  |
| 4+ | 660 (3.60%) | 657 (3.65%) | 3 (0.85%) |  |
| 5+ | 45 (0.25%) | 45 (0.25%) | 0 (0.00%) |  |
| Albumin:Creatinine Ratio (A:C) |  |  |  | <0.001 |
| 0 | 9,737 (53.05%) | 9,690 (53.83%) | 47 (13.28%) |  |
| 1+ | 7,337 (39.97%) | 7,066 (39.25%) | 271 (76.55%) |  |
| 2+ | 1,282 (6.98%) | 1,246 (6.92%) | 36 (10.17%) |  |

Note: Continuous variables are presented as median [interquartile range (IQR)]; categorical variables as n (%). Urinary sediment quantitative variables: cell-related indicators (cells/high-power field [HPF]), cast-related indicators (casts/low-power field [LPF]), other elements (units/HPF). Dipstick parameters were converted to ordinal variables (0 = negative, 1 = 1+, 2 = 2+, 3 = 3+, 4 = 4+, 5 = 5+). HPF = high-power field (400× magnification); LPF = low-power field (100× magnification). *P*-values were calculated using the Mann-Whitney *U* test for continuous variables and Chi-square test for categorical variables.
