# Supplementary material for: Development and validation of a routine urinalysis-based risk stratification model for IgA nephropathy in a multicenter real-world hospital population
Source: Front Nephrol. 2026 Aug 11;6:1826486. doi: 10.3389/fneph.2026.1826486 (PMC13503150; doi:10.3389/fneph.2026.1826486)
Supplement: Supplementary file 5 [file Table5.docx]

**Supplementary Table S2. Missing data summary for the 50 candidate routine urinalysis variables**

| **Variable** | **Missing observations** | **Missing rate within variable** | **Missing rate among all data cells** | **Imputation method** | **Retained in final model** |
| --- | --- | --- | --- | --- | --- |
| Coccilike bacteria | 35 | 0.19% | 0.004% | Median imputation | No |
