# Supplementary material for: Development and validation of a routine urinalysis-based risk stratification model for IgA nephropathy in a multicenter real-world hospital population
Source: Front Nephrol. 2026 Aug 11;6:1826486. doi: 10.3389/fneph.2026.1826486 (PMC13503150; doi:10.3389/fneph.2026.1826486)
Supplement: Supplementary file 6 [file Table6.docx]

**Supplementary Table S3. Bootstrap validation results for the primary 13-variable full urinalysis model**

| **Cohort** | **Apparent AUC** | **Bootstrap AUC Estimate (1000 resamples)** |
| --- | --- | --- |
| Training | 0.849 | 0.841 |
| Validation | 0.861 | 0.857 |
