# Supplementary material for: Development and validation of a routine urinalysis-based risk stratification model for IgA nephropathy in a multicenter real-world hospital population
Source: Front Nephrol. 2026 Aug 11;6:1826486. doi: 10.3389/fneph.2026.1826486 (PMC13503150; doi:10.3389/fneph.2026.1826486)
Supplement: Supplementary file 7 [file Table7.docx]

**Supplementary Table S4. Regression coefficients of the simplified 8-variable dipstick-based model**

| **Variable** | **β** | **SE** | **OR** | **95% CI** | **P value** |
| --- | --- | --- | --- | --- | --- |
| Intercept | -2.831 | 0.657 | 0.059 | 0.016–0.214 | <0.001 |
| Occult blood | 0.406 | 0.047 | 1.501 | 1.369–1.646 | <0.001 |
| Protein | 0.377 | 0.047 | 1.457 | 1.329–1.598 | <0.001 |
| Leukocyte esterase | -0.559 | 0.092 | 0.572 | 0.477–0.685 | <0.001 |
| Glucose | 0.166 | 0.039 | 1.181 | 1.095–1.275 | <0.001 |
| pH | -0.412 | 0.113 | 0.663 | 0.531–0.827 | <0.001 |
| Vitamin C | 0.407 | 0.073 | 1.502 | 1.303–1.732 | <0.001 |
| Ketone bodies | -0.737 | 0.261 | 0.479 | 0.287–0.798 | 0.005 |
| Specific gravity | -0.214 | 0.079 | 0.807 | 0.691–0.943 | 0.007 |

Note: The simplified model was developed as an implementation-oriented secondary model and was not intended to replace the primary full urinalysis model. Continuous variables were standardized using z-scores before model fitting.
